# From “Problem Trainees” to Collective Growth: A Self-Ethnographic Analysis of Tension and Change in Medical Education

**DOI:** 10.7759/cureus.103452

**Published:** 2026-02-12

**Authors:** Ryuichi Ohta

**Affiliations:** 1 Community Care, Unnan City Hospital, Unnan, JPN

**Keywords:** difficult encounter, education, emotions, family medicine, medical, organizational culture, professionalism, psychological safety, qualitative research, rural

## Abstract

Introduction

In medical education, trainees labeled as “problematic” are commonly framed as individuals with deficits in professionalism or competence. Such approaches may overlook the relational and organizational processes through which difficulty emerges. This study aimed to explore how “problem trainees” are constructed within training environments and how educators’ emotional experiences reflect broader organizational dynamics.

Methods

A self-ethnographic study was conducted based on longitudinal reflective documentation of seven complex educational encounters in a rural general medicine training program. Data included contemporaneous reflective notes, supervisory records, and team-based observations accumulated over several years. An iterative analytic process was used to identify recurring relational and organizational patterns, with attention to educator emotions, team interactions, and evolving educational practices.

Results

Five interrelated processes were identified. First, the label of “problem trainee” emerged relationally through accumulating frictions rather than isolated incidents. Second, educators experienced emotional turbulence, such as anger, avoidance, exhaustion, and self-doubt, which preceded overt organizational breakdown and signaled systemic strain. Third, psychological safety eroded through collective silence and avoidance, reinforcing deficit-based interpretations. Fourth, teams shifted from individual remediation toward collective holding, renegotiating shared professional norms and responsibilities. Finally, despite apparent stabilization, unresolved tensions persisted, highlighting the limits of educational intervention and the absence of definitive closure.

Conclusion

The emergence of “problem trainees” reflects relational and organizational processes rather than individual deficits. Educators’ emotional experiences serve as early indicators of systemic strain, and unresolved tensions may be an integral component of organizational learning. Recognizing and engaging with these dynamics may support more sustainable and reflective approaches to medical education.

## Introduction

Medical education has increasingly acknowledged the need to accommodate diverse medical trainees who are expected to develop into competent medical professionals despite differences in cognitive styles, mental health conditions, and physical traits [1,2]. In response to this diversity, contemporary medical education has emphasized inclusiveness and flexibility, aiming to broaden educational perspectives beyond standardized training models [3]. To achieve genuinely inclusive learning environments, medical education must adopt comprehensive educational approaches that recognize and respond to individual differences among trainees rather than treating them as deviations from a normative ideal [4].

General medicine, which addresses not only biomedical problems but also the psychological and social dimensions of health, is often considered particularly well positioned to engage trainees from diverse backgrounds [5]. Its emphasis on holistic care, contextual understanding, and interprofessional collaboration may create opportunities to support a broader range of learners within clinical training settings [6]. In this sense, fostering inclusivity within general medicine education has the potential not only to support trainee development but also to enrich academic practice and strengthen team-based clinical care [7].

Despite these aspirations, including trainees with diverse traits remains challenging in practice. Many medical education systems and organizational cultures were not initially designed for inclusive societies, and educators and healthcare professionals are often unprepared to respond to behaviors that deviate from implicit norms of professionalism, communication, or emotional regulation [8]. As a result, postgraduate medical trainees at various stages of residency training, including early, mid, and advanced phases, who struggle with punctuality, interpersonal relationships, emotional regulation, or communication styles may be labeled as “problematic.” These difficulties are typically recognized through patterns of interaction within the clinical learning environment, including engagement with supervising physicians, multidisciplinary staff, and team-based clinical workflows, and may generate tension within educational teams and disrupt clinical environments [9,10].

As a physician and medical educator leading a general medicine department in a rural hospital, I have encountered a subset of supervisory situations in which sustained relational tensions between trainees and the clinical team prompted repeated concern, dialogue, and organizational response. These encounters did not reflect routine variability among trainees but rather revealed recurring patterns in how educational teams interpreted, responded to, and adapted to behaviors perceived as difficult or destabilizing within the clinical learning environment. Over time, these experiences prompted critical reflection on how such tensions emerged and how educational and organizational practices evolved in response [11,12]. These encounters triggered conflicts not only between trainees and educators but also among team members, forcing the organization to confront its own implicit norms regarding fairness, professionalism, and safety. Rather than representing isolated incidents, these situations required repeated negotiation, adaptation, and reflection at both individual and collective levels.

From an academic perspective, these experiences motivated a critical examination of how medical education teams respond to trainees perceived as “problematic” and how such encounters can catalyze organizational learning and transformation [13]. This study employs an autoethnographic approach to analyze my experiences as an educator embedded within these tensions, focusing on how conflicts surrounding trainee inclusion prompted shifts in educational practices, team dynamics, and evaluative frameworks. By examining these processes reflexively, this paper aims to illuminate how challenges associated with trainee diversity can serve as opportunities for collective growth, offering transferable insights for medical educators seeking to build more inclusive and transformative training environments.

## Materials and methods

Study design

This study employed a qualitative autoethnographic design to examine the processes through which tensions arising from the inclusion of medical trainees perceived as “problematic” contributed to collective learning and organizational change within a general medicine training program [14,15]. Autoethnography enables systematic analysis of the researcher’s own lived experiences while situating them within broader social, cultural, and institutional contexts. This approach is particularly appropriate for exploring implicit norms, power relations, and emotional labor in medical education, which are often inaccessible through conventional observational methods [14,15].

Rather than aiming to generalize individual trainee behaviors, this study focused on how interactions between trainees, educators, and the clinical team revealed underlying assumptions about professionalism, fairness, and inclusion, and how these assumptions were negotiated and transformed over time.

Researcher positionality and reflexivity

The author (RO) is a practicing physician and head of a general medicine department at a rural hospital, with formal responsibility for trainee supervision, evaluation, and educational program management. This positionality entails inherent power asymmetries between the researcher and the trainees described in this study. Acknowledging this dual role as both educator and researcher, reflexivity was central to the research process.

Throughout the study, the author engaged in ongoing reflexive practice, systematically documenting personal emotional responses, including frustration, uncertainty, moral distress, and self-doubt, through contemporaneous reflective field notes and analytic memos. These reflections were examined longitudinally to explore how emotional responses influenced educational decisions, supervisory actions, and interpretive frameworks. Although reflexive accounts were not formally validated by an independent moderator, they were situated within institutional and organizational contexts through routine educational discussions with faculty colleagues and ongoing engagement in departmental educational governance. Ethical oversight was provided through review and approval by the Ethics Committee of Unnan City Hospital, Unnan, Japan (Approval code: 20190005). By explicitly situating the analysis within this positionality and institutional context, the study aimed to avoid retrospective rationalization and to foreground moments of ambiguity, failure, and ethical tension as legitimate sources of analytic insight.

Setting and context

This study was conducted in the general medicine department at Unnan City Hospital, a rural community hospital in Japan that serves as a training site for postgraduate medical residents within the national residency training system. Trainees enter the program through a national matching process based on trainee preferences and institutional selection procedures. The department emphasizes holistic patient care, interdisciplinary collaboration, and community-oriented practice, and accepts trainees with varied prior training environments, levels of clinical experience, and professional interests. In this context, “diverse backgrounds” refers to differences in educational trajectories, clinical exposure, and professional orientations, rather than to psychological or diagnostic categories.

During the study period, the department encountered a subset of trainees whose behaviors were perceived by supervising physicians and multidisciplinary staff as challenging existing educational and professional norms. These included difficulties with punctuality and attendance, recurrent interpersonal tensions within team-based clinical work, emotionally strained supervisory interactions, and communication patterns that disrupted established team workflows. These perceptions emerged through routine workplace-based supervision and team interaction, rather than formal psychological evaluation, and were documented in supervisory records and reflective field notes as part of ongoing educational practice. Rather than constituting isolated incidents, these encounters unfolded longitudinally and prompted repeated reflection and organizational response among educators and staff.

Such situations served as critical moments that triggered organizational reflection, revealing tensions between individual developmental needs, patient safety, and the sustainability of team functioning. In response, the department engaged in adaptive educational and organizational processes, including shifts in supervision practices, clarification of shared norms, and collective dialogue. The study period extended from April 2018 to January 2026, allowing for longitudinal observation of recurring patterns and organizational learning.

Trainee assessment and supervisory structure

The trainees described in this study were postgraduate medical residents participating in Japan’s nationally regulated residency training system. During their placement in the general medicine department, trainees were assessed primarily through workplace-based assessment conducted by supervising physicians with formal educational responsibilities. These assessments included longitudinal direct observation of clinical performance, evaluation of clinical reasoning and professionalism, review of clinical documentation, and multisource feedback from nurses and other multidisciplinary staff. Supervisors completed structured written evaluations as part of institutional and national training requirements.

Supervising physicians responsible for trainee evaluation were experienced clinicians who participated in faculty development activities, including training in clinical supervision, assessment practices, and educational feedback. Assessment processes were guided by national competency frameworks and institutional evaluation standards, which provided structured criteria for assessing trainee progression and performance.

Importantly, the trainees included in this study were not selected on the basis of formal examination failure or inability to progress within the training program. Most continued their training and met formal competency requirements. The analytic focus of this study was not academic performance per se, but rather relational and organizational tensions that emerged within workplace-based educational interactions, which are not fully captured by formal assessment outcomes.

Data sources

Multiple data sources were used to enhance analytic depth and reflexive rigor, consistent with a self-ethnographic approach. Data were accumulated longitudinally over the study period and reflected both contemporaneous documentation and retrospective reconstruction of educational practice.

Reflective Field Notes

The author maintained reflective field notes documenting educational encounters, emotional responses, supervisory decisions, and perceived team dynamics. Contemporaneous notes were recorded during periods of active supervision, capturing immediate reactions and situational interpretations. Retrospective field notes were reconstructed using institutional records, calendars, and personal archives to document earlier phases of departmental development. This process allowed for longitudinal tracing of relational and organizational patterns across different training cohorts.

Institutional Documents

Institutional documents were reviewed to contextualize individual episodes within broader organizational frameworks. These included educational policies, trainee evaluation forms, meeting minutes, and internal communications related to supervision and departmental operations. These materials provided insight into how expectations, norms, and responses evolved over time and how individual cases were situated within institutional decision-making processes.

Informal Feedback and Observational Insights

Informal verbal feedback from trainees, faculty members, nurses, and allied health professionals was incorporated to capture shifts in team perceptions and relational dynamics. These observations were limited to interactions during trainees’ active placement in the department and documented contemporaneously in reflective field notes. No attempt was made to formally collect or solicit reflections from trainees after their departure from the department to maintain appropriate professional boundaries and avoid ethical concerns related to supervisory power asymmetry. Accordingly, the analysis reflects relational and organizational dynamics as observed within the training context, rather than longitudinal trainee perspectives beyond their institutional placement.

Such feedback was recorded in anonymized form within reflective notes and treated as indicative of collective sense-making rather than as objective assessments. Observational insights from daily clinical practice further informed the analysis by documenting patterns of interaction, silence, and engagement across professional roles. Across all data sources, materials were treated not as objective representations of events but as situated accounts reflecting evolving interpretations and organizational meanings. Analytic attention was directed toward how narratives emerged, circulated, and stabilized within the department over time.

Case selection and operationalization of “problem trainee”

The seven encounters analyzed in this study were selected purposively from longitudinal reflective field notes and institutional records collected between April 2018 and January 2026. Inclusion criteria were defined to identify encounters that illuminated relational and organizational dynamics central to the study’s research question. Specifically, cases were included if they met all of the following conditions: (1) the encounter involved sustained relational tension extending beyond isolated incidents, (2) the situation prompted repeated supervisory attention, team discussion, or institutional response documented in reflective notes or organizational records, and (3) the encounter led to observable adjustments in supervision practices, team communication, or shared educational norms.

Encounters limited to routine educational challenges, transient misunderstandings, or situations resolved through single feedback interactions without broader organizational implications were excluded. The selected cases, therefore, represent analytically significant episodes characterized by longitudinal relational complexity and organizational impact, rather than a comprehensive enumeration of all difficult supervisory encounters during the study period.

Importantly, the designation “problem trainee” was not based on formal institutional classification, predefined behavioral thresholds, or retrospective labeling by the author alone. Instead, it was operationalized analytically as a relational construct, defined by the gradual emergence and circulation of concern among educators and multidisciplinary staff, as documented in supervisory records, informal feedback, and institutional communications. This operationalization reflects the study’s focus on how difficulty is socially and organizationally constructed rather than inherent to individual trainees.

The seven cases constitute a purposive subset selected for their analytic richness and their ability to illuminate recurring relational and organizational processes relevant to the research question, rather than a complete representation of all complex encounters during the study period.

Analytic approach

Data analysis followed an iterative inductive-deductive process consistent with self-ethnographic inquiry. Analysis was conducted by the author, who held dual roles as supervising educator and departmental leader. Reflexive attention was maintained throughout the analytic process to examine how positionality, emotional responses, and organizational responsibility shaped interpretation. Ethical oversight was provided through review and approval by the institutional ethics committee, and analytic rigor was strengthened through triangulation with institutional documents, longitudinal memoing, and ongoing engagement in departmental educational discussions.

Reflective field notes, institutional documents, and observational records were read repeatedly to identify critical incidents characterized by heightened relational tension, emotional disruption, or shifts in team functioning. Initial coding was conducted inductively, focusing on concrete interactions, emotional responses, and observable relational patterns. Examples of initial codes included “staff expressing concern indirectly,” “educator avoiding interaction,” and “uncertainty regarding supervision boundaries.” These codes were grounded in contemporaneous documentation and analytic memos.

Codes were then compared across cases and grouped into broader analytic categories representing shared relational processes, such as “informal circulation of concern,” “emotional withdrawal as organizational signal,” and “ambiguity in professional expectations.” Through iterative comparison and reflexive memoing, these analytic categories were synthesized into higher-level themes that captured recurring organizational dynamics.

For example, initial codes such as “staff avoiding direct feedback,” “private expressions of concern,” and “educator reluctance to engage” were clustered into the analytic category “collective avoidance,” which contributed to the broader theme “erosion of psychological safety and emergence of collective silence.” This progression from raw observation to thematic interpretation is illustrated in Table 1.

**Table 1:** Example of Coding Workflow and Thematic Development

Data excerpt	Initial code	Analytic category	Theme	Analytic memo logic
Several nurses mentioned discomfort but avoided raising it during formal meetings.	Indirect staff concern	Informal circulation of concern	Erosion of psychological safety and emergence of collective silence	Concern exists but cannot be voiced safely, indicating reduced psychological safety and relational avoidance.
I noticed myself postponing meetings with the trainee due to emotional exhaustion.	Educator avoidance	Emotional withdrawal	Educators’ emotional turbulence as an early signal of organizational strain	Educator avoidance reflects emotional burden and signals deeper systemic and relational strain.
Feedback conversations shifted from correcting the trainee to clarifying shared team expectations.	Shift from individual correction to team dialogue	Collective reframing of responsibility	Renegotiation of professional norms through collective holding	Educational difficulty reframed from individual deficit to shared organizational responsibility.

Subsequently, emerging themes were examined through theoretical lenses, including psychological safety, epistemic injustice, and transformative learning. Theory was introduced after inductive pattern identification and used interpretively rather than classificatorily. This abductive movement between empirical observation and theoretical interpretation enabled exploration of how individual encounters contributed to collective organizational learning.

Temporal mapping was employed to trace how similar relational tensions were interpreted and managed differently across phases of departmental development. Analytic memos documented shifts in interpretation and preserved analytic uncertainty as a meaningful feature of organizational learning rather than seeking premature resolution.

Ethical considerations

This study employed a self-ethnographic design based on the author’s professional experiences as a supervising physician and educator, along with anonymized institutional documentation and contemporaneous reflective field notes. Under applicable ethical guidelines in Japan, such research may qualify for exemption from full institutional review board review when it does not involve prospective participant recruitment, direct intervention, or use of identifiable personal data. Nonetheless, to ensure appropriate ethical oversight, the study protocol was submitted to and reviewed by the Ethics Committee of Unnan City Hospital, which independently evaluated the study design, confidentiality safeguards, and ethical considerations, confirmed its eligibility for exemption from full board review, and approved its conduct (Approval code: 20190005).

Given the author’s formal supervisory authority over trainees, particular attention was paid to minimizing risks of coercion, confidentiality breaches, and power asymmetry. No trainees or staff were prospectively recruited as research participants, and no individuals were contacted for research purposes. Data consisted solely of retrospective reflective field notes, anonymized institutional documents, and observational insights generated during routine educational practice. The ethics committee approved the use of these materials and determined that individual informed consent was not required because no identifiable personal information was included and no direct participant involvement occurred.

To protect confidentiality, all descriptions were carefully anonymized, and identifying details were modified or omitted to prevent individuals from being recognized. The analysis focused on organizational and relational processes rather than evaluating or diagnosing individual trainees. Reflexive attention was maintained throughout the study to avoid reinforcing deficit-based interpretations and to foreground systemic and contextual dimensions of educational tension. These safeguards ensured ethical integrity while allowing critical examination of organizational learning processes within medical education.

Trustworthiness and rigor

Triangulation was conducted across multiple internal data sources, including reflective field notes, institutional supervisory documentation, and contemporaneous feedback from multidisciplinary staff. Because the analytic focus was on organizational and relational dynamics within a specific clinical training environment, formal triangulation with external institutions, such as medical schools or prior training sites, was not undertaken. Access to external performance evaluations would have required identifiable trainee information and direct participant involvement, which was beyond the ethical scope and methodological intent of this self-ethnographic study.

To protect confidentiality and avoid deductive identification, detailed demographic, diagnostic, or performance-ranking information about individual trainees was intentionally not collected or reported. The analytic emphasis was not on individual characteristics such as age, sex, ethnicity, or diagnostic status, but on relational and organizational processes observed within the educational environment. This approach aligns with the study’s objective of examining how difficulty is constructed and managed within institutional contexts, rather than attributing difficulty to individual trainee traits.

## Results

Overview of results

The cases involved trainees with diverse professional backgrounds and personal circumstances, including differences in clinical experience, learning styles, mental health vulnerabilities, and social contexts. An overview of the included cases and their primary relational challenges is presented in Table 2. 

**Table 2:** Overview of Included Educational Cases and Relational Challenges This table summarizes the relational and organizational characteristics of the included educational encounters. To protect confidentiality and prevent deductive identification, detailed demographic, diagnostic, and performance-related information (e.g., age, sex, ethnicity, prior academic ranking, or medical diagnoses) is intentionally not reported. The study's analytic focus is on relational and organizational processes rather than on individual trainee attributes.

Case	Training stage	Primary relational challenge	Key source of tension	Organizational response	Persistent unresolved tension
Case 1	Early training	Authoritative communication and resistance to supervision	Patient safety concerns and hierarchical conflict with multidisciplinary staff	Escalated feedback, shared supervision, and organizational boundary-setting	Long-term suitability for training and trust restoration
Case 2	Early–mid training	Interprofessional conflict and a critical stance toward other physicians	Breakdown of collegial relationships and team cohesion	Explicit feedback, norm clarification, reinforcement of help-seeking	Balance between critical thinking and collaborative practice
Case 3	Mid training	Inconsistent attendance and reliability	Perceived unfair workload distribution and erosion of team morale	Explicit expectations, shared norms, ongoing dialogue	Extent and limits of accommodation for individual needs
Case 4	Mid training	Misalignment between learning style and clinical pace	Delayed patient management and communication gaps	Dialogical adjustment of learning strategies, team-level awareness	Optimal timing and depth of early educational intervention
Case 5	Early training	Perfectionism and difficulty adapting to uncertainty	Clinical inefficiency and emotional distress	Reframing of expectations, longitudinal dialogue, and peer support	Sustainability of growth under high self-imposed standards
Case 6	Early–mid training	Communication style causing interpersonal friction	Psychological safety and trust within the team	Consistent feedback, shared team approach, and role clarification	Ongoing tension in managing neurodiversity in training
Case 7	Mid–late training	Emotional instability affecting interprofessional collaboration	Team fatigue and boundary ambiguity	Increased dialogue, team restructuring, and reflective support	Distinguishing adaptation from habituation over time

**Figure 1:**
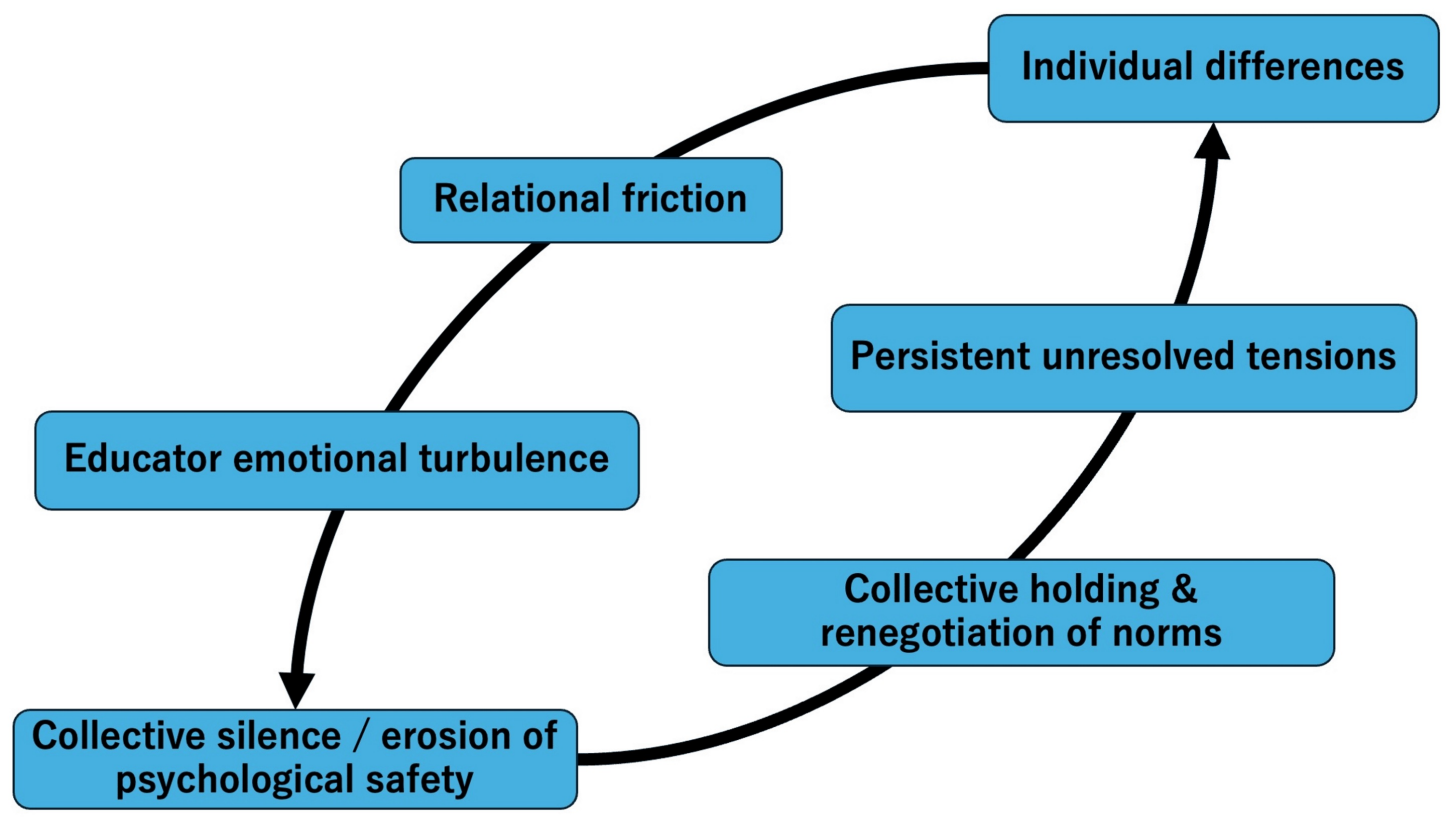
Relational and Organizational Processes Underlying the Emergence of “Problem Trainees” The figure illustrates how individual differences interact with relational dynamics and organizational responses, leading to collective learning without definitive resolution.

Across seven extended educational encounters in a rural general medicine training program, trainees who were eventually labeled as “problematic” did not initially present as uniformly difficult or deficient. Instead, difficulties emerged gradually through repeated interactions involving trainees, educators, multidisciplinary staff, and institutional expectations. Analysis revealed that these tensions were shaped not only by trainee behaviors but also by organizational and relational factors, including variability in supervisory approaches, differences in communication norms across professional roles, and structural constraints within the clinical environment. These findings suggest that the emergence of difficulty reflected systemic and relational processes rather than solely individual trainee characteristics. Five interrelated themes were identified: (1) the relational construction of the “problem trainee,” (2) educators’ emotional turbulence as an early signal of organizational strain, (3) erosion of psychological safety and the emergence of collective silence, (4) renegotiation of professional norms through collective holding rather than individual correction, and (5) the persistence of unresolved tensions despite apparent stabilization. These themes represent not linear stages but overlapping processes that recurred across cases with differing personal backgrounds, clinical competencies, and psychosocial contexts (Figure 1).

Theme 1: the relational construction of the “problem trainee”

Across all included cases, the label of “problem trainee” did not arise from a single defining incident or objective assessment of competence. Rather, it developed gradually through the accumulation of relational friction within everyday clinical practice. Early signals were often ambiguous and indirect, conveyed through comments such as “staff seem uncomfortable,” “communication feels difficult,” or “working with this trainee is exhausting.” These expressions rarely identified specific behaviors at first, but instead reflected diffuse unease within the team.

In several cases, trainees initially made favorable impressions. Some were described as highly motivated educators, clinically enthusiastic, or deeply committed to patient care. However, as clinical demands increased, these same traits became sources of tension when they interacted with unspoken organizational norms. For example, strong confidence and decisiveness were initially welcomed but later reinterpreted as arrogance when accompanied by dismissive attitudes toward nurses or resistance to multidisciplinary input. Educational enthusiasm was similarly reframed as problematic when trainees delegated procedures without adequate supervision or failed to recognize their own limits in high-risk situations.

Importantly, the escalation of concern was rarely triggered by isolated behaviors. Rather, meaning was assigned through repetition and circulation. Reports from nurses, administrative staff, and fellow trainees were accumulated through informal conversations, handovers, and supervisory discussions. Over time, these fragmented impressions coalesced into a shared narrative in which the trainee was increasingly referred to as “difficult,” “unsafe,” or “unprofessional,” even when specific incidents varied across observers.

Contextual factors played a critical role in this interpretive process. The same behaviors-such as assertive communication, persistent clinical reasoning, or slow and meticulous patient management-were interpreted differently depending on team workload, staffing levels, and prior relational history. In high-pressure contexts, behaviors that deviated from expected rhythms of care were more readily framed as problematic. Once a trainee was positioned outside the perceived norm, subsequent actions were more likely to be interpreted through a deficit-based lens, reinforcing the categorization regardless of intent or improvement.

This process revealed that “problematic” status was not a stable attribute of individual trainees, but a relational outcome produced at the intersection of personal styles, team expectations, and organizational culture. Crucially, once the label became embedded, it shaped future interactions by narrowing interpretive flexibility. Behaviors that might otherwise have been viewed as developmental or situational were instead understood as confirmation of an already established identity, making re-negotiation of meaning increasingly difficult. In this sense, professional identity emerged not as a fixed individual trait but as a socially constructed and continually negotiated outcome shaped by relational and organizational contexts.

Theme 2: educators’ emotional turbulence as an early signal of organizational strain

As the supervising educator and departmental leader, my emotional responses to these situations were neither stable nor linear. Initial encounters with new trainees were often marked by anticipation, professional curiosity, and a sense of responsibility for their growth. However, as relational tensions accumulated, these feelings gradually shifted toward irritation, frustration, and moral distress. These emotional changes did not occur suddenly; rather, they intensified alongside repeated episodes of misalignment between trainees’ behaviors and the team's expectations.

In several cases, anger emerged in response to behaviors perceived as arrogant, dismissive, or unsafe, particularly when trainees failed to seek supervision in high-risk clinical situations or responded defensively to feedback. At other times, my dominant response was avoidance. I noticed myself postponing difficult conversations, limiting contact to essential interactions, or delegating supervisory responsibilities in order to reduce emotional burden. This withdrawal was accompanied by a sense of fatigue and emotional depletion, especially when similar conflicts recurred despite repeated feedback efforts.

These emotional reactions were closely tied to concerns about patient safety and professional accountability. Situations in which trainees placed patients at risk, by proceeding without appropriate consultation, failing to recognize limitations, or disregarding team input, provoked intense ethical unease. I found myself questioning not only the trainee’s readiness but also my own adequacy as an educator and leader. Doubts emerged regarding whether the existing supervision structures were sufficient, whether expectations had been communicated clearly, and whether I had misjudged the trainee’s capacity at earlier stages.

Importantly, these emotions were not experienced as purely personal or idiosyncratic. They consistently coincided with moments when the team’s educational frameworks proved insufficient to contain emerging complexity. Feelings of frustration and exhaustion appeared before explicit breakdowns in team communication, such as collective silence or overt conflict. In this sense, emotional turbulence preceded and anticipated visible organizational strain.

In retrospect, these emotional responses functioned as early warning signals rather than individual weaknesses. Anger, avoidance, and self-doubt marked points at which individual remediation was no longer adequate and collective reconsideration of educational norms, supervision practices, and shared responsibilities became necessary. Educators’ emotions thus served as diagnostic data, revealing the limits of existing systems and signaling the need for organizational-level learning.

Theme 3: erosion of psychological safety and the emergence of collective silence

As relational tensions accumulated, psychological safety within teams gradually eroded. Rather than manifesting as open conflict, this deterioration was most clearly expressed through silence. Multidisciplinary staff and fellow trainees became increasingly hesitant to voice concerns directly, particularly in shared clinical spaces such as rounds or case conferences. Concerns were more often expressed indirectly, through informal comments, private messages, or offhand remarks that suggested unease without naming its source.

A recurring feature of this phase was the pervasive sense that “no one says it directly, but the atmosphere has become heavy.” Team members appeared to recognize the tension collectively, yet lacked a shared space or language to address it openly. As a result, communication shifted from explicit dialogue to avoidance-based strategies. Participation in clinical discussions declined, feedback was softened or withheld, and joint rounds were sometimes shortened or strategically avoided.

In several cases, staff chose to distance themselves from the trainee rather than engage in potentially conflictual dialogue. This distancing took multiple forms, including limiting collaboration to task-based interactions, refraining from offering guidance, or quietly reassigning responsibilities. While these actions reduced immediate discomfort, they also narrowed opportunities for mutual understanding and learning. Trainees, in turn, often perceived this withdrawal as rejection or hostility, further deepening relational strain.

Importantly, collective silence did not neutralize tension; instead, it amplified the “problem trainee” label. Unresolved concerns continued to circulate informally, accumulating without opportunities for clarification or contextualization. In the absence of open dialogue, behaviors were increasingly interpreted through pre-existing narratives, reinforcing deficit-based assumptions and reducing interpretive flexibility. Silence thus became an active force in sustaining problematic dynamics rather than a passive absence of speech.

The erosion of psychological safety, therefore, functioned both as a consequence and a driver of relational strain. As safety diminished, teams became less able to engage in corrective or reflective dialogue, which in turn entrenched avoidance, misinterpretation, and emotional withdrawal. This cyclical process marked a critical transition point at which individual-focused educational responses became insufficient, setting the stage for subsequent collective renegotiation of norms. Concurrently, educators experienced increasing moral distress as their professional responsibility to support trainee development came into tension with concerns about team functioning, patient safety, and organizational sustainability, further reinforcing patterns of emotional withdrawal and collective silence.

Theme 4: renegotiation of professional norms through collective holding

When repeated individual corrective efforts failed to resolve ongoing tensions, educational practices gradually shifted from trainee-centered remediation toward collective holding. Rather than continuing to focus exclusively on modifying the trainee’s behavior, teams began to engage in explicit dialogue about shared expectations regarding professionalism, communication, supervision, and mutual accountability. This shift marked a qualitative change in how difficulties were framed and addressed.

Collective holding involved acknowledging that misalignment did not reside solely within the trainee but emerged from interactions among trainees, educators, and institutional structures. Educators openly recognized their own limitations, including uncertainty about appropriate supervisory boundaries, delayed intervention, or inconsistent messaging. At the same time, teams began to articulate contextual factors shaping trainees’ behaviors, such as workload intensity, developmental stage, mental health vulnerabilities, and personal or family-related stressors. These factors were not treated as excuses but as realities that required collective awareness and adaptation.

In several cases, teams made implicit norms explicit. Baseline expectations-such as timely communication regarding absences or delays, respect for multidisciplinary roles, transparent help-seeking in uncertain clinical situations, and shared responsibility for patient safety-were articulated as collective commitments rather than individualized demands. Importantly, these norms were framed as applicable to all team members, including senior physicians, thereby reducing ambiguity and perceived inequity.

Through sustained dialogue and shared responsibility, teams gradually developed greater tolerance for uncertainty and difference. Situations that had previously been interpreted as evidence of individual failure were reframed as collective challenges requiring ongoing negotiation. This reframing allowed partial restoration of trust and collaboration, even when difficulties persisted. Rather than achieving resolution through correction, teams learned to stabilize relationships by collectively holding tension, enabling continued clinical work and educational engagement despite unresolved complexity. In this process, encounters initially framed as individual trainee difficulty functioned as catalysts for leadership reflection and organizational learning, revealing how psychological safety, moral responsibility, and professional identity were negotiated at the level of the supervising educator and the broader clinical team.

Theme 5: persistent unresolved tensions and the limits of educational intervention

Despite the adaptive processes described above, not all tensions were resolved through collective holding and renegotiation of norms. In several cases, concerns regarding long-term suitability for training, equitable distribution of clinical workload, and the emotional burden placed on specific team members continued to surface intermittently. These concerns did not disappear but instead became less visible as teams stabilized daily operations.

Educators remained ambivalent about where to draw boundaries between inclusion and patient safety, as well as between supporting individual trainees and sustaining organizational functioning. Questions persisted regarding how much accommodation was appropriate, when continued support risked overburdening others, and whether stability was being maintained at the cost of silent strain. These uncertainties were particularly salient in cases involving repeated absences, uneven performance, or ongoing interpersonal friction.

Notably, periods of apparent harmony sometimes gave rise to new unease. Reduced conflict raised questions about whether mutual understanding had deepened or whether team members had simply adapted by lowering expectations, avoiding engagement, or suppressing frustration. In some instances, what appeared as calm reflected habituation rather than resolution, leaving educators uncertain whether underlying issues had been meaningfully addressed.

These ongoing ambiguities underscored the limits of educational intervention. Organizational learning did not culminate in clear closure or definitive solutions; instead, it involved managing persistent uncertainty over time. Maintaining awareness of unresolved tension, rather than attempting to eliminate it, became a pragmatic stance that allowed teams to continue functioning while remaining attentive to potential re-emergence of strain. In this sense, unresolved tension itself became part of the educational landscape, shaping how teams understood growth, responsibility, and the boundaries of support. Rather than representing failure, the capacity to acknowledge, contain, and work within unresolved tension emerged as a critical organizational competence, enabling sustained educational practice in complex and uncertain clinical environments.

## Discussion

Summary of the study

This self-ethnographic study examined how trainees labeled as “problematic” emerged within a rural general medicine training program, focusing on relational and organizational processes rather than individual deficits. Across seven extended educational encounters, difficulties did not arise from isolated incidents or stable personal characteristics but were constructed through repeated interactions among trainees, educators, multidisciplinary staff, and unspoken institutional norms.

Five interrelated processes were identified. First, the “problem trainee” label developed relationally through accumulating frictions and informal sense-making. Second, educators’ emotional turbulence, manifesting as anger, avoidance, exhaustion, and self-doubt, preceded overt organizational breakdown and functioned as an early signal of systemic strain. Third, psychological safety eroded through collective silence and avoidance, which paradoxically reinforced deficit-based interpretations. Fourth, teams shifted from individual correction to collective holding, renegotiating professional norms and shared responsibility. Finally, despite apparent stabilization, unresolved tensions persisted, highlighting the limits of educational intervention and the absence of definitive closure.

Together, these findings suggest that difficulty in medical training emerges through the interaction between individual trainee characteristics and relational and organizational contexts. While individual differences may contribute to these challenges, the present analysis highlights how educational teams collectively interpret, respond to, and manage such situations. In this sense, difficulty is not solely an individual deficit but a relational phenomenon that reveals how educational systems respond to complexity, vulnerability, and uncertainty.

Comparison with other studies

Previous medical education literature has largely framed “problem trainees” in terms of remediation, professionalism lapses, or competency deficits, often emphasizing individualized assessment and correction [16-18]. While such approaches provide useful tools for addressing observable behaviors, they tend to underemphasize the relational and organizational contexts in which difficulties arise [19,20].

More recent work on professional identity formation, faculty emotional labor, and psychological safety has begun to acknowledge the role of social and organizational factors in training environments [21,22]. Studies examining remediation processes have noted the emotional burden placed on educators and the challenges of balancing patient safety with learner support [23,24]. However, these discussions are often treated as secondary considerations rather than analytic focal points.

The present study extends this literature by positioning educators’ emotional responses and team-level dynamics as central data rather than background context. In contrast to models that assume resolution through successful remediation, this analysis demonstrates that organizational learning often proceeds without closure [25,26]. Stabilization may occur through collective holding and norm renegotiation, even as fundamental tensions remain unresolved [27]. This perspective aligns with sociocultural and relational theories of learning, emphasizing process over outcome and uncertainty over definitive solutions.

Strengths of the study

A key strength of this study lies in its use of self-ethnography to access aspects of medical education that are often difficult to capture through conventional methodologies. By foregrounding the educator’s lived experience, including emotional responses and ethical unease, this approach illuminated early signals of organizational strain that might otherwise remain unarticulated. The longitudinal, case-diverse nature of the documentation allowed for the identification of recurring relational patterns across trainees with differing backgrounds, learning styles, and psychosocial contexts. Rather than presenting isolated anecdotes, the study traced processual dynamics that unfolded over time, enhancing analytic depth and transferability.

Additionally, intentionally minimizing individual trainee characteristics reduced the risk of reinforcing deficit-based interpretations. This analytic choice supported the study’s relational focus and maintained attention on organizational processes rather than personal pathology. Through reflexive self-ethnographic analysis, the educator’s own emotional experiences-including uncertainty, frustration, and moral distress-were treated not as sources of bias to be eliminated, but as meaningful analytic data that illuminated otherwise inaccessible relational and organizational dynamics within medical education.

Limitations

This study has several limitations. First, it reflects the perspective of a single educator within a specific rural general medicine context, which may limit generalizability. The findings are not intended to represent universal training environments but to offer transferable insights through thick description. Second, the retrospective nature of the analysis introduces the possibility of recall bias and post hoc sense-making. Although contemporaneous reflections and institutional documentation informed the analysis, interpretations were inevitably shaped by subsequent reflection. Third, the absence of direct trainee or multidisciplinary staff narratives constrains the multiplicity of perspectives represented. While this limitation is inherent to self-ethnography, it also underscores the need for future studies incorporating dialogical or multi-voice approaches. Additionally, because data collection was limited to the author’s observations and contemporaneous workplace interactions, trainee perspectives after departure from the department were not systematically collected. This may limit the representation of trainees’ evolving interpretations and introduce the possibility of a partial perspective inherent to educator-centered autoethnographic analysis.

## Conclusions

This study suggests that trainees perceived as “problematic” emerge from interactions among individual characteristics, team dynamics, and organizational context rather than solely from individual deficits. While conducted in a rural Japanese training program, the findings highlight transferable mechanisms, including the relational construction of difficulty, the role of educator emotional responses as early indicators of systemic strain, and the importance of team-level interpretation and response. These findings have practical implications for medical education. Educator emotional responses may serve as early warning signals prompting reflective dialogue, while structured team-based discussions and shared supervisory responsibility may help restore psychological safety and support both trainee development and organizational stability. Importantly, educational tensions may not always be fully resolved; the capacity to recognize and manage unresolved tension represents a key component of organizational learning. Attending to relational dynamics may help training programs foster more adaptive, reflective, and sustainable learning environments.
